# Supplementary material for: Interpretation of Multivariate Association Patterns between Multicollinear Physical Activity Accelerometry Data and Cardiometabolic Health in Children—A Tutorial
Source: Metabolites. 2019 Jul 2;9(7):129. doi: 10.3390/metabo9070129 (PMC6680435; doi:10.3390/metabo9070129)
Supplement: Supplementary file 1 [file metabolites-09-00129-s001.pdf]

metabolites

MDPI

Article

Interpretation of Multivariate Association Patterns between Multicollinear Physical Activity Accelerometry Data and Cardiometabolic Health in Children—A Tutorial

Eivind Aadland [1,*], Lars Bo Andersen [1], Geir Kåre Resaland [2] and Olav Martin Kvalheim [3]

1 Department of Sport, Food and Natural Sciences, Faculty of Education, Arts and Sports, Campus Sogndal, Western Norway University of Applied Sciences, 6856 Sogndal Norway
2 Center for Physically Active Learning, Faculty of Education, Arts and Sports, Western Norway University of Applied Sciences, Campus Sogndal, 6856 Sogndal, Norway
3 Department of Chemistry, University of Bergen, 5007 Bergen, Norway
* Correspondence: eivind.aadland@hvl.no; Tel.: +47-5767-6086

Supplemental

Table S1. Predicted change in the cardiometabolic health composite score (1 SD) by changes in decentiles of time spent in 3000-3999 and 7000–7999 cpm.

	**Decentiles of 7000–7999 cpm**										
	1	2	3	4	5	6	7	8	9	10	Mean
	Predicted change in the cardiometabolic health composite score (1 SD)										
Boys	1.08	0.75	0.57	0.36	0.17	0.00	−0.21	−0.46	−0.81	−1.48	0.00
Girls	0.79	0.52	0.35	0.25	0.09	−0.02	−0.16	−0.34	−0.49	−0.99	0.00
	Decentiles of 3000–3999 cpm										
	1	2	3	4	5	6	7	8	9	10	Mean
	Predicted change in the cardiometabolic health composite score (1 SD)										
Boys	0.38	0.27	0.19	0.13	0.07	0.00	−0.07	−0.15	−0.28	−0.52	0.00
Girls	0.44	0.35	0.29	0.20	0.17	0.10	0.03	−0.03	−0.12	−0.26	0.00
